# Spirituality in pain medicine: A randomized experiment of pain perception, heart rate and religious spiritual well-being by using a single session meditation methodology

**DOI:** 10.1371/journal.pone.0203336

**Published:** 2018-09-07

**Authors:** Anja Sollgruber, Helmar Bornemann-Cimenti, Istvan-Szilard Szilagyi, Andreas Sandner-Kiesling

**Affiliations:** 1 Department of Anaesthesiology, Pain and Intensive Care Medicine, University of Medicine, Graz, Austria; 2 Department of Special Anaesthesiology, Pain and Intensive Care Medicine, University of Medicine, Graz, Austria; Emory University, School of Public Health, UNITED STATES

## Abstract

The aim of this study is to investigate different effects on pain perception among randomly assigned volunteers practicing meditation compared to a relaxation condition. The study examines whether participants of the experimental conditions (meditation versus relaxation) differ in the change of pain perception and heart rate measurement and in religious and spiritual well-being after an intervention. Method: 147 volunteers (long-term practitioners and novices) were randomly assigned to the experimental conditions with a headphone guided 20-minute single session intervention. The change in their pre- and post-intervention pain perception was measured using Quantitative Sensory Testing and Cold Pressor Testing (CPTest), their stress-level was compared by monitoring heart rate, and their religious and spiritual well-being by using the Multidimensional Inventory for Religious/Spiritual Well-Being (MI-RSB48). Additionally, dimensions of the Brief Symptom Inventory (BSI) measured the psychological resilience of the participants; pain and stress experience, and the state of relaxation and spirituality experience were assessed. Five persons were excluded due to failure in measuring the heart rate and 29 participants had to be excluded because of high values on the BSI. Results: The meditation group showed an increase in their pain tolerance on the CPTest and a decrease in their pain intensity for heat after the experimental condition, in contrast to the relaxation group. Futhermore, the meditation group showed a higher level of religious spiritual well-being (MI-RSB48 Total score) as well as in the sub-dimensions General Religiosity, Forgiveness, and Connectedness after the experimental condition, compared to the relaxation group. Our data is consistent with the hypothesis that meditation increases pain tolerance and reduces pain intensity, however, further work is required to determine whether meditation contains similar implications for pain patients.

## Introduction

Pain, and especially chronic pain, is a serious health problem that affects millions of people. For these people pain is a near-everyday experience and is considered a major clinical, social, and economic problem in communities around the world [1,2]. Traditional pain therapies do not always relieve pain and rarely improve quality of life [1]. Consequently, recent research on pain relief focuses on the efficiency and effectiveness of complementary approaches [3]. Pain is said to protect the body from damage caused by overload [4]. The International Association for the Study of Pain (IASP) defines pain as "an unpleasant sensory and emotional experience associated with actual or potential tissue damage" [5], thus, precluding an idea of pain as a purely sensory experience [6]. Already in the 1960s, the Gate Control Theory, a complex psychobiological pain model by Mellzack and Wall, led to a far-reaching concept change which Ronald Melzack revolutionized again in 1999. Through a series of neural networks, the brain acts as an active part of the pain process, thus contributing to the pain occurrence. When activated, these networks integrate information or schemata from multiple areas in the body, including emotional, physical, and cognitive areas (eg cultural factors, past experiences, personality traits and situational features of the pain experience), to modulate the subjective sense of pain in terms of increasing or decreasing it [7]. Newer definitions state that "pain is a distressing experience associated with actual or potential tissue damage, with sensory, emotional, cognitive and social components" [8]. These components of the pain are, to a large extent, subjective [9]. As a result, the pain is a personal, internalized and multi-level experience and therefore the feeling of pain of each person differs [10,11]. Consequently, studies have shown that the severity of pain is more related to psychological and physical well-being than the location of the pain [12,13].

Recent studies now support the salutogenic role of religiosity and spirituality and religiously-spiritual interventions in pain management [14–27]. Spiritual approaches to pain management can take a variety of forms. These can include different approaches from prayer to participation in religious services, spiritual rituals, and meditation [28,29]. Some of these approaches are explicitly religious, some spiritual or secular, but their roots can be found in religious and spiritual traditions [28,29]. For this reason, a definition of spirituality is required. Spirituality is understood as a form of belief that goes beyond belief in the sense of a particular religious community. Spirituality can, but does not have to, be linked to a belief system and rather describes a non-traditional, non-denominational form of belief [30]. Some authors use the term spirituality as synonymous with religiosity, but they emphasize personal beliefs, regardless of a religious confessional community [31].

### Meditation and pain

Earlier meditation research describes the process of applying certain techniques to bring about a particular state of consciousness in which there is a profound shift in the relationship to thoughts and emotions, resulting in greater clarity, perspective, objectivity and ultimately equanimity [32]. From today's point of view of research, meditations to delineate specific psychological processes based on traditional meditation texts and modern neuroscientific conceptions are divided into two broad categories: Focused Attention (FA) and Open Surveillance (OM) Meditation [33]. The term “meditation” refers to mental and emotional practices from a number of cultural contexts including those of Christianity and Islam, yet is most frequently applied to those originating in the Eastern spiritual traditions of India, Tibet, China, and Japan [34]. Meditation has been adopted in western countries both as a spiritual practice and a mind–body therapeutic intervention [35].

In recent decades, empirical meditation research as well as mindfulness research and the use of these techniques in clinical practice have steadily expanded. It has been well documented that meditation and mindfulness-based interventions (MBI’s) are associated with several positive outcomes, such as a decrease in psychiatric symptomatology (eg, anxiety, depression) or an increase in positive states (eg, well-being, positive emotions) as well as physical well-being [36–38]. Metastudies report that mindfulness meditation improves pain [39] depression symptoms and quality of life [38–40]. Furthermore other studies reported, it seems that spiritual meditation has a unique additional effect of reducing the negative effect [20,27]. Studies show that access to spiritual resources is associated with improved pain tolerance in acute pain [20], chronic pain [23,38,41,42] arthritic pain [42], for migraine pain [20,27] and experimental and clinical pain [43]. A series of studies with a sample size of 22 participants [44] investigated the effects of mindfulness meditation on electrically induced pain stimulation. In the first experiment, the research group used a 3-day mindfulness meditation (20min/day). The pain value was measured before and after the intervention. In addition, the degree of mindfulness and anxiety were assessed. The participants’ assessment of pain for "low" and "high" electrical stimulation decreased significantly after meditation training, as did the sensitivity to pain measured by the stimulus thresholds (pain thresholds). A second experiment showed that a 'mathematical distraction task' reduced high pain values, but not a relaxation condition. There was no reduction in pain sensitivity in these participants. A third experiment directly compared the effects of meditation with a 'mathematical distraction task' and a relaxation condition. The results showed significant effects in both the meditation and 'mathematical distraction task' conditions, but not in the relaxation group. Consistent with what was observed in the first experiment, these participants also showed a decrease in pain sensitivity after meditation training. The changes in mindfulness and in the assessment of anxiety suggest that the pain-reducing effect of meditation is related to reduced anxiety and the improved ability to focus on the present moment.

It has been shown that positive spiritual coping ability in different stress situations is extremely helpful; it influences pain management and has an impact on the progression of pain chronification [22,45]. Spirituality and Spiritual Intervention are associated with lower pain intensity, pain perception, reduced pain frequency and pain duration [18,46–58]. It is suggested that resource mobilization also helps the patient to accept the disease or pain, thus affecting pain perception and, consequently, pain and suffering [26,50–52].

In addition to psychological and religious spiritual well-being, stress plays an essential role in pain, can contribute to pain, affects pain management [12,13], and is therefore a robust predictor of chronic pain [53]. Studies have shown that meditation also has a significant positive effect on the cardiovascular system [16] as well as a demonstrable reduction in physiological and psychological distress [37,54,55]. According to newer studies, the "mindfulness stress buffer" resulting from meditation reduces stress, has an impact on promoting resilience and, consequently, on a wide range of physiological health outcomes. It seems that this "stress buffer" helps in the acceptance of experiences and the resulting ability to regulate emotions can be understood as stress-coping strategies that promote stress resistance [56].

Thus, meditation and mindfulness-based interventions can affect a number of different areas that affect pain sensation and pain tolerance. The positive psychological and physiological effects mentioned above suggest that a meditation technique can be helpful to people who are in pain, and also support them in dealing with the resulting stress. Research in recent decades has shown that psychological, social and behavioral factors can modulate the perception of pain and also affect the transition from acute to chronic pain [47,53,58]. Further studies suggest that spirituality is of relevance to mental and physical parameters of health and disease, since spirituality can influence and interlink social, psychological, and even biological aspects of human life [59].

Nevertheless, data on the effectiveness of meditation, especially in the area of pain, is still sparse. Early meditation research compared meditation to other clinical strategies, including visualizations, self-hypnosis, biofeedback, progressive muscle relaxation, and autogenic training [60,61]. The results of several research studies are disputed or inconsistent [62–66] or suffered from considerable methodological and conceptual limitations (eg. unrepresentative sample sizes, few novices, suboptimal controls of adherence compliance, type and duration of meditation) as well as effect modifiers (eg. age, gender of the meditator, stress level, mental health,) usually being neglected in studies and thus limiting the generalization of results [38,67,68].

While previous studies consistently observed positive psychic and physical salutogenic outcomes among meditation techniques [69], few studies have attempted to scientifically investigate in detail their influence on pain in terms of pain perception, pain intensity, pain tolerance and pain processing with an experimental design and unravel the different underlying mechanisms.

### Aim of this research

The aim of this research is to gain information on how a practice of meditation in a single session relates to pain experience and religious spiritual well-being. The underlying study examines the effect of pre- and post- random interventions (guided meditation versus relaxation) on changes in the various individual pain sensations; The perception, intensity, tolerance, and cardiovascular reactivity to these stimuli and the sense of religious spiritual well-being were scientifically studied in an experimental design. A reduced pain sensation and a higher pain tolerance is expected, after a meditation session as well as a increase of religious spiritual well-being.

Consequently, the focus of the recent study is on two different areas. On the one hand, the range of physiological data (pain detection thresholds, pain intensity, pain tolerance) and the cardiovascular reactivity (heart rate, HR) and on the other hand, on the subjective reactions of the participants to pain as well as the intervention by psychological and religious spiritual variables. Physiological variables provide detailed objective information about pain perception and pain tolerance of the participants, whilst cardiovascular reactivity (HR) serves as an objective indicator of response to the stress experienced. The subjective perception of pain and stress during the pain testing as well as the subjective perception of relaxation and spirituality in relation to the intervention were recorded. Furthermore, the religious spiritual well-being, assessed by means of a questionnaire before and after the intervention, was also recorded.

## Materials and methods

### Participants

A total of 147 volunteers (94 females, 53 males, all Caucasian), aged from 18 to 69 years (mean age = 41.5, SD = 12.5), were tested. Participants were recruited through flyers and public disclosures (such as in shops, at bus stops, on bulletin boards, in large companies in the capitals Graz, Vienna, and Linz), and assessment took place from October 2016 to January 2017.

This study was approved by the Ethics Committee of the Medical University of Graz EK28-461ex15/16, conducted in accordance with the Declaration of Helsinki, and, furthermore, every participant gave written informed consent.

### Questionnaire measures

#### Meditational practice

All participants answered the question whether they practice meditation at regular intervals (no/yes). Those who answered this question with "yes" were asked how often they practice (daily/ once a week/ once a month).

#### Psychological measures

All participants completed the Brief Symptom Inventory [70] (BSI) which was given after participants consented to join the study. The BSI is a short version of the Short Symptom Checklist [71] (SCL 90 R)—a well-established, self-report survey which comprises 53 items designed to offer rapid screening of the symptoms of psychological disorders and assesses the nine primary symptom dimensions: Somatization, Obsessive-Compulsive, Interpersonal Sensitivity, Depression, Anxiety, Hostility, Phobic Anxiety, Paranoid Ideation and Psychoticism.

#### Visual analogue scales (VAS)

The study included eight different visual analogue scale. Participants were asked to rate their 1) subjective religious faith, 2) their dimension of religious faith, 3) their dimension of spirituality and 4) their attachment to an ecclesiastical community.

For a manipulation check of the physiological measurements participants were asked to rate their subjective experience of 5) stress and 6) pain, and for the intervention, participants were asked to rate their subjective experience of 7) relaxation and 8) spirituality while using their technique [16]. The visual analogue scales for stress and pain were administered at two intervals. The scores of all visual analogue scales ranged from 0 (“totally disagree”) to 100 (“totally agree”).

#### Spirituality measures

The Multidimensional Inventory for Religious/Spiritual Well-Being [72] (MI-RSB 48) consists, in total, of 48 items and six subscales, each measured with eight items: Hope, Immanent, Forgiveness, Experience of Sense and Meaning, Hope Transcendent, General Religiosity and Connectedness. Participants had to answer on a six-point rating scale ranging from 1 (“totally disagree”) to 6 (“totally agree”). The MI-RSB 48 was administered at two intervals.

### Physiological measures

#### The quantitative sensory tests (QST)

The standardized quantitative sensory testing (QST) was developed by the German Research Network on Neuropathic Pain (DFNS) [73] and represents a standardized extension of the clinical neurological sensitivity test. It is a non-invasive, well-reproducible method for the measurement of sensor technology, and pain perception and sensory perception functions in standardized form [74]. The study includes thermal stimuli and consists of three tests that measure six different parameters. These are thermal detection thresholds for the perception of cold (CDT) or warmth (WDT), thermal pain thresholds for the perception of cold (CPT) and heat pain (HPT), as well as cold and heat pain values for the detection of pain intensity. By applying defined thermal stimuli to the body surface, perception, pain thresholds and pain intensity are measured. For standardized and reproducible applications, QST provides valid and reliable clinical and scientific information [75]. The QST study was performed using the Thermal Sensory Analyzer (TSA, Medoc, Israel).

#### Cold Pressor Test (CPTest)

The quantitative sensory testing was extended by a cold-pressor test (CPTest). A cold-water test is a common laboratory approach for measuring pain tolerance as well as the pain threshold [76,77]. This serves to measure both physical and psychological responses with regard to pain. In the recent study, the CPTest consists of a two-degree Celsius cold-water bath, into which the participant places his hand up to the wrist until it becomes unbearably painful. The time is measured as the seconds which the participant´s hand remains in the water bath and thus depicts the pain tolerance.

#### Heart rate

Measurement of heart rate and heart rate variability has become a common tool in the clinical domain [78]. The heart rate is the number of heart beats per minute and is most frequently used in psychophysiology as an indicator of cardiovascular events. Almost every change in physical and psychological state is accompanied by heart rate changes [76,77]. The heart rate was recorded by means of a Polar training computer. This consists of a transmitter and a receiver; the latter can be a bracelet receiver or a mobile phone by using an app. This training computer records the number of heart beats per minute and provides valid and reliable clinical and scientific information [78,79]. The transmitter provided for this purpose is attached around the chest by means of an elastic band and measures the heart rate based on the electrocardiogram principle. This ensures an accurate and reliable indication of the heart rate (heart beats per minute). To measure the heart rate, the "Polar Bluetooth Smart Heart Rate Chest Transmitter", a bracelet receiver and the app "Ithlete pro" was used.

### Experimental intervention

The experimental intervention is a single session methodology. Both the meditation as well as the relaxation conditions were individual sessions and had the same procedures, the same duration and settings. The participants were randomly assigned to two experimental conditions:

#### Meditation group

The meditation group were headphone-guided during their 20-minute intervention. They were given written instructions about how to relax physically, to sit in any position they like, as long as they would not fall asleep. They were instructed to "make yourselves calm", focus on their meditation and pay attention to their breathing for the duration of the time. If the participants felt themselves losing focus, they were advised just to refocus and continue with their intervention. "It is essential that you do not sleep while meditating or think about stressful things but focus on this meditation. However, if you do become distracted, concentrate on the meditation again and continue". At the end of the 20-minute session they heard the sentence: "Please end your relaxation and wait for further instruction".

"Now you have 20 minutes to relax with your eyes closed and to turn to meditation. In this, different spiritual techniques are used. 'Spiritual' can, but does not have to be understood in the Christian denominational sense.

If you find the term "divine" or "sacred" unsuitable, you can replace it with Higher Power, Allah, JHWH, Tao, Brahman, Prajna, All-One, Great Power, Everything, Source, or whatever you conceive it to be.

For the meditation, please take a sitting position which allows you to sit comfortably and relaxed for 20 minutes. This meditation will be played to you by means of a tape and at the end of these 20 minutes you will hear the words "Please end your meditation now, open your eyes and turn your attention outwards again.". This is the indication that the "time is up". Please open your eyes again and wait for the next instruction.

It is essential that you do not sleep while meditating or think about stressful things, but focus on this meditation. If, however, you notice that you have become distracted, don’t worry; just concentrate on the meditation again and continue to do so. If you have any questions or anything is unclear, please ask your questions now.

When everything is clear and you are ready, please take your comfortable, relaxed position, close your eyes, focus on your breathing and start to relax."

#### Meditation

There are many definitions of meditation, but it was not until the Delphi study [80] that a sufficient consensus definition and taxonomy were developed. The developed meditation makes use of these approaches and can be divided into three areas:

1. At the beginning of the meditation, the eyes are closed and attention is paid to the breathing rhythm. This is supported by the inhalation of symbols such as the Sun's Light, which are associated with creative power [81].
2. The examiner directs his full attention towards himself and a place of rest within himself.
  Only after the mind has become accustomed to directing its attention to objects, will further techniques be used.
3. The mantra "I am loved, protected and guided, embedded in the big picture" should help the study participants to focus on one single item.

Emotional excitement is enhanced when one succeeds in focusing their undivided attention on one item [81]. The mantra is intended to support this.

The developed meditation is a kind of Taoist meditation which has many similarities to Hindu and Buddhist systems but is less abstract than contemplative traditions. Though mantras are usually associated with Hindu and Tibetan Buddhist practices, Taoists have also employed them for many millennia [82]. The primary hallmark of this meditation is the generation, transformation, and circulation of internal energy. A combination of concentrative meditation (visualizing a divine or a sacred symbol which is associated with creative power) and open monitoring types of meditation are involved, as well as breathing and body awareness in the sense of mindfulness.

#### Relaxation group

The relaxation group was given written instructions about how to relax physically, to sit in any position they would like, as long as they would not fall asleep. The relaxation group was given the same instructions as the meditation group regarding their posture of physically comfortable positions while in eyes-closed relaxation. But unlike the meditation group, the relaxation group was not given explicit instructions regarding how to mentally occupy themselves during these 20 minutes of eyes-closed rest-time. They were just told to " calm yourselves", to pay attention to their breathing and not to fall asleep or think about stressful things: "It is essential that you do not sleep during this 20-minute relaxation time or think about stressful things but concentrate on the relaxation. However, if you do become distracted, focus on the relaxation again and continue with the relaxation". During their 20-minute intervention they were headphone-guided with an 'empty tape'. At the end of the 20-minute session they heard the sentence: "Please end your relaxation and wait for further instruction".

“You now have 20 minutes to relax with your eyes closed. Please take a position that allows you to sit comfortably and relaxed for 20 minutes. During the relaxation you will be played an empty tape and at the end of these 20 minutes you will hear the words "Please end your relaxation session now, open your eyes and turn your attention outwards again". This is the indication that the "time is up".

Please open your eyes again and wait for the next instruction.

It is essential that you do not sleep during this 20 minutes relaxation time or think about stressful things, but turn your attention to this relaxation exercise. If, however, you notice that you have become distracted, don’t worry; just focus on the relaxation again and continue to do so.

If you have any questions or anything is unclear, please ask your questions now.

When everything is clear and you are ready, please take your comfortable, relaxed position, close your eyes and start to relax”.

### Procedure

After arrival at the laboratory, the participants`consent for the study was obtained before they were asked to sit quietly for 5 minutes. Sensors were connected for the heart rate measurements. Afterwards, participants completed a set of questionnaires assessing demographics, religious and spiritual (MI-RSB 48, VAS subjective religious faith, VAS dimension of religious faith, VAS dimension of spirituality, and VAS attachment to an ecclesiastical community) as well as current psychological state (BSI). After the baseline, an assessment of the physiological state measurements took place. This included heart rate (HR), cold detection threshold (CDT), warmth detection threshold (WDT), cold pain threshold (CPT), heat pain threshold (HPT) as well as the pain intensity for cold and for heat. Participants then performed a cold pressor test, a heart rate measurement, and rated the pain and stress experience on a VAS. After this baseline period, the participants were randomly assigned to one of two experimental conditions (the relaxation group or the meditation group), and the 20 minutes experimental condition, relaxation or meditation, took place. Both groups were headphone-guided during their 20-minute intervention. They were given written instructions. After the experimental condition, the participants rated their state of relaxation and spirituality experience on a VAS. The post-physiological measurements included again: heart rate, cold detection threshold, warmth detection threshold, cold pain threshold, heat pain threshold, pain intensity for cold and for heat. Participants then performed a cold pressor test, a heart rate measurement took place, and the participants rated their pain and stress experience on a VAS. After this, participants completed a final questionnaire assessment (MI-RSB 48).

### Statistical analysis

Categorical data was analyzed by means of the chi square test. Group differences in numerical variables were analyzed by using the t-test for independent samples. In the case of heterogeneous variances, Welch’s correction for the t-test was applied. In order to measure the effect size, the eta square coefficient was computed.

## Results

### Sample composition

Five persons were excluded due to failure in measuring the heart rate and 29 participants had to be excluded because of high values on the BSI (more than two standard deviations above the mean score of the normative sample). Therefore, the remaining sample consisted of 113 persons (75 females, 38 males) aged from 19 to 69 years with a mean age of 42.7 (SD = 11.9). Fifty-seven persons were randomly assigned to the relaxation group and 56 participants were assigned to the meditation group. The two groups did not differ statistically significantly in gender (*Chi*^*2*^(1) = 2.33, *p* = .127), age (*t*(111) = -0.29, *p* = .773), practicing of meditation at regular intervals (*Chi*^*2*^(1) = 1.65, *p* = .200), and frequency of practicing meditation (*Chi*^*2*^(2) = 3.22, *p* = .200).

### Manipulation check

A statistically significant difference between the groups was found in spirituality experience of their technique (relaxation group: M = 45.86, SD = 33.85; meditation group: M = 68.95, SD = 24.63; t(102.36) = -4.15, *p* < .001). No statistically significant group difference was found in the subjective rating of how relaxing each technique was (relaxation group: M = 84.98, SD = 24.48; meditation group: M = 91.30, SD = 20.50; t(111) = -1.49, *p* = .140).

### Baseline characteristics

As shown in Table 1, there was no statistically significant difference between the groups on any of the variables. All effect sizes ranged from 0 to .03, indicating very small differences between the groups.

**Table 1:** Baseline characteristics of physiological and questionnaire measurements by group.

	Relaxation group		Meditation group			
	M	SD	M	SD	*p*	Eta^2^
Physiological measurements						
Cold detection threshold	29.68	1.39	29.58	1.61	.732	.00
Warmth detection threshold	33.87	1.14	34.19	1.63	.232	.01
Cold pain threshold	7.69	7.11	6.06	6.08	.192	.02
Heat pain threshold	45.94	3.06	46.85	2.97	.111	.02
Cold Pressor Test sec.	19.98	11.36	24.56	21.10	.156	.02
Pain intensity cold	42.05	27.78	50.54	25.44	.093	.03
Pain intensity heat	57.79	25.19	61.71	26.84	.424	.01
Heart rate	66.93	8.52	68.55	9.88	.351	.01
Subjective stress	20.46	22.73	28.86	29.20	.091	.03
Subjective pain	38.28	29.28	46.04	31.01	.174	.02
Psychological measurements						
BSI Somatization	0.41	0.47	0.38	0.36	.723	.00
BSI Obsessive-Compulsive	0.64	0.40	0.65	0.48	.891	.00
BSI Interpersonal Sensitivity	0.54	0.42	0.46	0.40	.307	.01
BSI Depression	0.26	0.32	0.27	0.33	.907	.00
BSI Anxiety	0.41	0.31	0.38	0.38	.587	.00
BSI Hostility	0.44	0.37	0.44	0.41	.935	.00
BSI Phobic Anxiety	0.18	0.24	0.13	0.22	.219	.01
BSI Paranoid Ideation	0.47	0.47	0.37	0.36	.199	.01
BSI Psychoticism	0.27	0.25	0.18	0.27	.064	.03
General Religiosity	29.47	11.90	29.63	9.65	.941	.00
Forgiveness	38.44	6.96	37.29	7.28	.391	.01
Hope Immanent	40.77	4.35	39.48	4.25	.114	.02
Connectedness	32.19	10.06	29.80	8.33	.172	.02
Hope Transcendent	36.77	5.77	35.23	7.32	.217	.01
Experience of Sense and Meaning	39.70	5.35	39.23	5.62	.650	.00
Total Score	217.35	30.06	210.66	28.10	.224	.01
Subjective religious faith	54.56	30.88	53.33	24.60	.815	.00
Dimension of religious faith	44.40	29.59	41.61	25.88	.594	.00
Dimension of spirituality	57.96	25.76	50.38	25.38	.118	.02
Attachment to an ecclesiastical community	22.19	25.22	28.55	25.70	.187	.02

### Changes from pre- to post-measurements

In order to evaluate differences between pre- and post-measurement, change scores were computed. Each change score was calculated as post-measurement minus pre-measurement (baseline). Group differences in the change scores were evaluated by means of the t-test for independent samples.

#### Physiological measurements

The pain tolerance on the cold pressure test was statistically significantly increased, with a medium effect size, in the meditation group, compared to the relaxation group. As shown in Table 2, the mean duration of time the participants‘ hand was submersed in water was higher in the meditation group after the experimental intervention. Furthermore, the pain intensity for heat decreased statistically significantly in the meditation group after the experimental condition (Table 2). Neither in the detection threshold for warmth and cold nor in the pain threshold for cold and for heat statistically significant group differences were found after the experimental condition (Table 2). In addition, there was no statistically significant change in heart rate (Table 2).

**Table 2:** Change in physiological and questionnaire measurements by group.

	Relaxation group		Meditation group			
	M	SD	M	SD	*p*	Eta^2^
Physiological measurements						
Cold detection threshold	0.04	1.37	0.10	1.56	.804	.00
Warmth detection threshold	-0.40	0.88	-0.46	1.17	.751	.00
Cold pain threshold	-1.45	7.27	-1.21	4.40	.835	.00
Heat pain threshold	1.25	2.64	1.34	2.22	.837	.00
Cold Pressor Test sec.	-0.12	5.42	2.70	5.86	.010	.06
Pain intensity cold	-4.32	12.70	-8.46	14.93	.114	.02
Pain intensity heat	-3.33	10.73	-9.48	14.16	.010	.06
Heart rate	-2.48	5.63	-3.95	4.95	.149	.02
Psychological measurements						
Subjective stress	0.35	19.22	-5.70	26.20	.164	.02
Subjective pain	0.67	20.09	-5.91	25.16	.127	.02
Religious spiritual measurements						
General Religiosity	-0.02	3.34	1.66	4.51	.027	.04
Forgiveness	0.38	3.26	2.25	3.31	.003	.08
Hope Immanent	0.61	2.68	0.59	3.16	.974	.00
Connectedness	-0.04	3.04	1.61	4.19	.019	.05
Hope Transcendent	1.39	2.62	0.77	3.02	.245	.01
Experience of Sense and Meaning	-0.82	4.06	-0.02	2.79	.225	.01
Total Score	1.50	9.13	6.86	9.01	.002	.08

#### Spirituality measures

There was a statistically significant increase in religious spiritual well-being (MI-RSB48 Total score) as well as in the subdimensions General Religiosity, Forgiveness, and Connectedness, with small to medium sized effects (Table 2) after the experimental condition.

#### Subjective experience of stress and pain (VAS)

There was no statistically significant change in the subjective experience of stress and pain on the VAS over the whole pain condition (Table 2).

## Discussion

This study was performed in order to examine the effect of a headphone -guided mindfulness single session meditation compared to a relaxation condition on different pain sensations (cold and heat pain stimulus), pain perception, pain intensity, pain tolerance, consequently on the pain processing, on the cardiovascular reactivity to these stimuli as well as on the religious spiritual well-being.

The recent study focused on two different areas: on the one hand on the physiological data on pain experience (pain detection thresholds, intensity, tolerance) and the cardiovascular reactivity (heart rate) and on the other hand, on the participants`subjective responses to the pain, as well as to the intervention. Physiological variables provided detailed objective information on pain perception and on the pain tolerance of the participants, whilst cardiovascular reactivity served as an objective indicator of the response to the experienced stress. In addition, the subjective perception of pain and stress in the pain testing as well as the subjective perception of relaxation and spirituality in relation to the intervention were assessed, and the religious spiritual well-being determined through questionnaires before and after the intervention.

In summary, the study supports most of the underlying hypotheses in this project. Through a 20-minute single session meditation, the meditation group was able to develop a higher pain tolerance in the cold pressor test, as well as a lower pain sensation or a lower pain intensity in the heat pain value. In addition, the meditation group, in comparison to the relaxation group, developed a greater sense of religious spiritual well-being and thus had more religiously spiritual coping resources available.

### Mindfulness meditation with religiously spiritual content—Pain and heart rate reactivity

At approximately the same cold and warmth detection threshold and pain threshold for cold and heat, as well as at approximately the same subjectively experienced stress (VAS) and objective stress levels (heart rate) the meditation group was able to develop greater pain tolerance during a 20-minute single sesssion meditation compared to the relaxation group. Furthermore, the meditation group reported lower pain intensity with respect to heat pain compared to the relaxation group, but there was neither a difference nor a slight tendency in the intensity of cold pain. This means that participants of both groups perceived both the warmth and the cold stimulus almost equally quickly, had an approximately equal pain threshold for cold as well as for heat, and these stimuli also appeared to be equally painful and stressful (as measured by VAS and HR). However, those who used the meditation technique seemed to have a higher tolerance or endurance to cold-induced pain and, in addition, appeared to develop less sensitivity to the heat pain. Thus, the meditation group did not change the sensitivity to warmth or cold—how quickly they perceive a change in temperature—nor to pain—from when they felt a stimulus painful. It also seems that the subjective perception as well as the stress level is not altered by the use of a meditation technique, but merely its evaluation and management of that pain, and consequently how well they tolerated that level of pain. It does not seem that this altered state of consciousness is a dissociated state in which the participant reduces the focus on the body so that he can withstand discomfort longer, as this would also be accompanied by altered sensitivity or altered cold and warmth detection thresholds. It rather seems to be an altered process of awareness that allows people to reframe experiences and better cope with or tolerate adverse conditions and thus endure discomfort for longer.

Based on the data, the differences between the groups are not in the lower sensitivity, in terms of sensory perception of pain, but probably in the in the cognitive and emotional processing of pain, which with a low sensitivity or a better coping with the affective and cognitive dimensions, consequently goes along with the sensory dimensions of the pain. The data thus suggests that meditation leads to a psychic or cognitive process of re-evaluation, which consequently alters the pain evaluation and thus the pain tolerance and not the pain perception or pain thresholds.

Similar to other studies that investigated the effects of spiritual practices on experimentally induced pain, the practice of meditation in this study did not alter the pain intensity with respect to cold pain [16,42]. However, meditation had an effect on how well the subjects tolerated the pain level for cold and how intensively they rated a heat pain. Thus, in this study there appears to be a qualitative difference in the assessment of pain intensity (based on the assessment of pain value) for cold and heat pain.

Although there were differences in pain tolerance between the groups, the expected corresponding significant differences in cardiac rate reactivity / stress reactivity to pain did not appear. These results are also consistent with the subjectively experienced stress, which was perceived approximately equally in both groups. Thus, neither statistically significant group differences with regard to the subjectively experienced stress (VAS) nor due to the objective data (heart rate) could be found. This suggests that the existing differences in pain tolerance as well as pain intensity should be sought in the altered inner state and the resulting cognitive emotional evaluations, and not in the different stress levels of the groups.

Meditation thus offers a method of pain management, to improve pain tolerance and reduce the pain intensity of a cold pain. It appears that this method brings, with relatively little effort, a remarkable level of pain tolerance for people, regardless of the degree of religiosity or spirituality of the participants and regardless of the experience in meditation.

### Religiously spiritual (well-being) and mindfulness meditation

The efficacy findings of the meditation group brought important insights regarding the religiously spiritual feeling of well-being. In the manipulation check, the meditation group reported a significant difference in the subjectively experienced spirituality (VAS). They experienced more spiritual intensification during the 20-minute meditation and reported more spiritual experiences than the relaxation group. Futhermore, the meditation group showed a higher level of religious spiritual well-being (MI-RSB48 Total score) as well as in the sub-dimensions General Religiosity, Forgiveness, and Connectedness after the experimental condition, compared to the relaxation group. A closer analysis of the data and subscales revealed an increase of positive feelings such as optimism, hope, security, contentment, peace and compassion as well as trust in finding support with God or something divine. As a result, participants of the meditation group had more religious spiritual coping resources available after the meditation. The assumption suggests that the physiological benefits of meditation lie in the qualitative experience or in the qualitative difference.

Meditation can thus influence a number of different areas that have an impact on pain sensation and pain tolerance, or areas that are associated with low sensitivity to the affective and sensory dimensions of pain. The assumption suggests that the physiological benefits of meditation lie in the qualitative experience or in the qualitative difference.

### Limitations and future directions

Although the 20-minute guided single session meditation was related pain tolerance as well as pain intensity in the expected direction, this study still has some limitations.

Since this is a pilot study, the participant population was a healthy one. Ending the study at this point means that it lacks follow-up data as well as data on patients suffering from pain. While the recent study provided promising results after just 20 minutes single session guided meditation, it nevertheless leaves open the question of whether these benefits would be sustained over time, or whether these effects could be found in pain patients. Future studies should focus on pain patients and / or on follow-up data to investigate the duration of efficacy of the effects. In order to gain information about the influences of spirituality on pain, follow-up studies should also use a three-subject check group survey (a secular mindfulness condition, the religious spiritual mindfulness condition, and the relaxation condition). Furthermore, the participants of the secular mindfulness condition and the participants of the religious spiritual mindfulness condition should be kept blind to which is the experimental intervention. To consider possible effects of the self-selection bias, it would be helpful to increase the interest in the study or in the topic.

Religiosity and spirituality often overlap in content, so they should be examined on the one hand as related to one another, and on the other hand, as independent concepts [83]. However, an even more detailed analysis and a distinction between religiosity and spirituality are needed. Thus, a more accurate survey of the subjectively experienced experiences of religiosity, spirituality, pain and relaxation would be recommended, for example by means of short standardized interviews. In the manipulation check, a distinction with regard to subjectively experienced religiosity as well as subjectively experienced spirituality could also be made.

Different approaches would allow for more complex pathway analyses that would help us deepen our understanding of the relationship between meditation religious spiritual well- being stress and, its connection to pain and its processing.

The results of these studies suggest that meditation interacts with other secular stress management strategies within emotion, behavior regulation and positive reappraisal. The relationship between spiritual support and indicators of subjective well-being has proved complex in several studies [84]. There remains substantial potential for further research and ongoing dialog aimed at developing deeper, more integrated understandings of the spiritual, psychological, biological effects and effects of meditative practice on pain management.

## Conclusions

Pain, and especially chronic pain, is a serious health problem that affects millions of people. Traditional pain therapies do not always relieve pain and rarely improve quality of life. It seems that a 20-minute meditation has profound effects within a short time. This means that meditation can create a double benefit: First, pain is better tolerated by meditation and perceived less intensely. Second, meditation leads to a greater sense of religious-spiritual well-being and more positive religious spiritual coping resources. A summary of all the results suggests that the increased pain tolerance and reduced pain intensity of the single session meditation is related to a more resourceful inner state and consequently, resulting in better coping strategies.

The meditation thus offers a recognized and validated method for changes in pain tolerance and pain intensity (for heat). The results suggests that meditation, regardless of whether religious or spiritual, is already an integral part of the person's system of values, can be used successfully in pain therapy with little effort and has a positive effect on religious spiritual well-being.

As a result, improved pain tolerance, less pain sensation, and increased religious spiritual coping resources would help people continue their day-to-day activities. In turn, this would be beneficial to their mental health and quality of life. Meditation can thus influence a number of different areas that have an impact on pain sensation and pain tolerance, or that are associated with low sensitivity to the affective and sensory dimensions of pain.

Thus, the information obtained from the present study suggests that meditation can promote both physical and mental religious spiritual health. However, additional studies are needed to determine whether meditation contains similar implications for pain patients and what effects it has when this technique is practiced over several days, weeks or months.

## Supporting information

S1 File Studies flow chart. (DOCX)

S2 File Instructions of the relaxation and meditation condition. (DOCX)

S3 File Raw data file. (XLS)
