# Supplementary material for: Spirituality in pain medicine: A randomized experiment of pain perception, heart rate and religious spiritual well-being by using a single session meditation methodology
Source: PLoS One. 2018 Sep 7;13(9):e0203336. doi: 10.1371/journal.pone.0203336 (PMC6128533; doi:10.1371/journal.pone.0203336)
Supplement: S1 File — (DOCX) [file pone.0203336.s001.docx]

**Spirituality in pain medicine**

**A randomized experiment of pain perception, heart rate and religious spiritual well-being by using a single session meditation methodology**

Fig 1: Studies flow chart

**Contact, consent**

**Initial questionnaire assessment**

MIRSB - 48, BSI, sociodemographic data

exclusion

**Heart rate (HR) baseline survey**

**QST measurement 1, cold pressor test 1 and heart rate**

**information on the subjective experience of pain and stress**

**randomized allocation to**

**single session meditation**

**randomized allocation to**

**relaxation**

**subjective experience of relaxation and spirituality during the intervention**

**QST measurement 2, cold pressor test 2 and heart rate**

**information on the subjective experience of pain and stress**

**Final questionnaire assessment**

MIRSB - 48
