# Supplementary material for: Spirituality in pain medicine: A randomized experiment of pain perception, heart rate and religious spiritual well-being by using a single session meditation methodology
Source: PLoS One. 2018 Sep 7;13(9):e0203336. doi: 10.1371/journal.pone.0203336 (PMC6128533; doi:10.1371/journal.pone.0203336)
Supplement: S2 File — (DOCX) [file pone.0203336.s002.docx]

**Instruction for relaxation**

You now have 20 minutes to relax with your eyes closed. Please take a position that allows you to sit comfortably and relaxed for 20 minutes. During the relaxation you will be played an empty tape and at the end of these 20 minutes you will hear the words "Please end your relaxation session now, open your eyes and turn your attention outwards again". This is the indication that the "time is up".

Please open your eyes again and wait for the next instruction.

It is essential that you do not sleep during this 20 minutes relaxation time or think about stressful things, but turn your attention to this relaxation exercise. If, however, you notice that you have becom distracted, don’t worry; just focus on the relaxation again and continue to do so.

If you have any questions or anything is unclear, please ask your questions now.

When everything is clear and you are ready, please take your comfortable, relaxed position, close your eyes and start to relax.

**Instruction for meditation**

Now you have 20 minutes to relax with your eyes closed and to turn to meditation. In this, different spiritual techniques are used. Spiritual can, but does not have to be understood in the Christian denominational sense. If you find the term „divine“ or „sacred" unsuitable, you can replace it with Higher Power, Allah, JHWH, Tao, Brahman, Prajna, All-One, Great Power, Everything, Source, or whatever you conceive it to be.

For the meditation, please take a sitting position which allows you to sit comfortably and relaxed for 20 minutes. This meditation will be played to you by means of a tape and at the end of these 20 minutes you will hear the words "Please end your meditation now, open your eyes and turn your attention outwards again". This is the indication that the time is up. Please open your eyes again and wait for the next instruction.

It is essential that you do not sleep while meditating or think about stressful things, but focus on this meditation. If, however, you notice that you have become distracted, don’t worry; just concentrate on the meditation again and continue to do so.

If you have any questions or anything is unclear, please ask your questions now.

When everything is clear and you are ready, please take your comfortable, relaxed position, close your eyes, focus on your breathing and start to relax.
